# Animal models of lung cancer: Phenotypic comparison of different animal models of lung cancer and their application in the study of mechanisms

**DOI:** 10.1002/ame2.70028

**Published:** 2025-05-19

**Authors:** Zixuan Yang, Xianbin Zhao, Lili Tan, Pingxinyi Que, Tong Zhao, Wei Huang, Dejiao Yao, Songqi Tang

**Affiliations:** ^1^ School of Traditional Chinese Medicine Hainan Medical University Haikou China; ^2^ School of Pharmacy Chengdu University of Traditional Chinese Medicine Chengdu China; ^3^ School of Basic Medical Sciences Chengdu University of Traditional Chinese Medicine Chengdu China; ^4^ Oncology Department II Hospital of Chengdu University of Traditional Chinese Medicine Chengdu China

**Keywords:** animal models of lung cancer, chemical induction methods, gene editing mouse models, lung cancer grafts, transplantation models

## Abstract

Lung cancer has one of the highest rates of incidence and mortality worldwide, making research on its mechanisms and treatments crucial. Animal models are essential in lung cancer research as they accurately replicate the biological characteristics and treatment outcomes seen in human diseases. Currently, various lung cancer models have been established, including chemical induction models, orthotopic transplantation models, ectopic transplantation models, metastasis models, and gene editing mouse models. Additionally, lung cancer grafts can be categorized into two types: tissue‐based and cell‐based grafts. This paper summarizes the phenotypes, advantages, and disadvantages of various induction methods based on their modeling techniques. The goal is to enhance the simulation of clinical lung cancer characteristics and to establish a solid foundation for future clinical research.

## Graphical abstract

Grafts used to construct lung cancer animal models come from various sources, primarily cell lines, patient‐derived tumor tissues, and circulating tumor cells. Based on the sources of grafts and research objectives, lung cancer animal models can be categorized into several types: chemical‐induced models, orthotopic transplantation models, heterotopic transplantation models, metastasis models, and gene‐edited models. These models are crucial for lung cancer research, as they can simulate various disease phenotypes, including cell proliferation, invasion and metastasis, epithelial‐mesenchymal transition (EMT), cell cycle regulation, gene mutations, apoptosis, and immune infiltration. These models serve as powerful tools for exploring the pathogenesis of lung cancer, identifying new therapeutic targets, and evaluating drug efficacy.

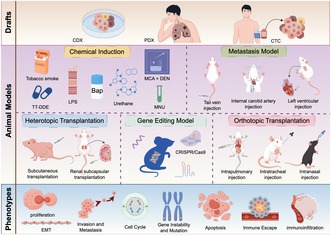

## 1 INTRODUCTION

Lung cancer is one of the most common malignant tumors worldwide, affecting the bronchus, alveoli, and other lung tissues.^1^ In 2022, nearly 2.5 million new cases of lung cancer were diagnosed, with over 1.8 million deaths worldwide, making it the most prevalent cancer in terms of both morbidity and mortality.^2^ Factors such as smoking, environmental pollution, and occupational exposure substantially elevate the risk of developing lung cancer.^3^, ^4^, ^5^ The emergence of lung cancer is characterized by genetic harm, mutations, and a decline in cellular functionality, reflecting a complex and multi‐stage journey. Currently, lung cancer treatment primarily involves surgery, radiotherapy, chemotherapy, targeted therapy, and immunotherapy, among other approaches. In this paper, the animal models of lung cancer – chemical induction, transplantation models, and gene editing mouse models – are summarized, and the advantages and disadvantages of different modeling methods are discussed (Table 1). Lung cancer transplantation models are categorized based on the inoculation site into orthotopic, ectopic, and metastasis models. Additionally, lung cancer grafts are classified into two categories: tissue grafts and cellular grafts. This aims to guide future animal experiments in lung cancer research, enhance the simulation of clinical lung cancer manifestations, and establish a foundation for subsequent clinical studies.

**TABLE 1:** Phenotypic comparison in animal models of lung cancer.

Type	Strain	Mouse age	Reagent	Methods	Phenotypes	References
Chemical induction						
Smoke‐induced lung cancer	Adult mice	8–12 weeks	Cigarette smoke	Exposed to cigarette smoke for 2 weeks	1–5	[6, 7]
NNK‐induced lung cancer	Albino Sprague Dawley	10 weeks	NNK	1.5 mg/kg three times weekly for 20 weeks, s.c.	1–3, and 6	[8]
	A/J mice	5–6 weeks		50 mg/kg for 4 weeks, i.p.	1–3, and 6	[9]
Urethane‐induced lung cancer	Albino Wistar	6 weeks	Urethane	1000 mg/kg, three consecutive i.p. injections separated by 48 h each week for 12 weeks	1, 3, 4, and 6	[10]
	C57BL/6	6–8 weeks		1 g/kg in 200 μL PBS, i.p.	1 and 5	[11, 12]
Bap‐induced lung cancer	A/J mice	5–6 weeks	Bap	1 mg /7 days for 4 weeks, i.p.	1, 3, and 7	[9]
	Swiss albino mice	7–8 weeks		50 mg/kg, twice weekly for four consecutive weeks, orally	1, 8, and 9	[13, 14]
MNU‐induced lung cancer	KrasLSL‐G12D mice and wild‐type mice	7–12 weeks	MNU	50 mg/kg for 20 weeks, i.p.	1 and 8	[15]
	FVB‐Trp53+/− mice and wild‐type mice	6 weeks		50 mg/kg for 26 weeks, i.p.	1 and 8	[16]
TT‐DDE‐induced lung cancer	ICR mice	6 weeks	TT‐DDE	24 mg/kg for 8 weeks, ITLI.	1, 3, and 6	[17]
MCA/DEN‐induced lung cancer	Wistar rat	6 weeks	MCA and DEN	10 mg MCA and 10 μL DEN instilled into the left lobe at the 15, 35, 55, 65, and 75 days	1 and 8	[18, 19, 20]
LPS‐induced lung cancer	A/J mice	5–6 weeks	LPS AND NNK	3 mg NNK and 5 μg LPS for 10 weeks, i.p.	1, 4, and 5	[21]
	C57BL/6 mice	5 weeks	LPS AND Bap	150 mg/kg NNK (i.p, biweekly for 4 weeks) and 0.25 mg/kg LPS (IN, weekly for 12 weeks)	1 and 4	[22]
Orthotopic transplantation						
Intrapulmonary injection	SD rats	—	3,4‐benzopyrene	Treated twice at a 2‐week intervals with 2 mg 3,4‐benzopyrene (dissolved in 0.2 mL corn oil) through intrapulmonary injection	1 and 6	[23]
	C57BL/6 mice	4–6 weeks	LLC‐luc	1.2 × 10^6^ cells/mL in a 1:1 ratio of PBS mixed with Matrige, Inject into the lung	1, 5–7	[24]
Intranasal injection	C57BL/6 and BALB/c nude mice	5–8 weeks	LLC and NCI‐H460	LLC (1 × 10^5^ to 1 × 10^7^ cells) or NCI‐H460 (5 × 10^6^) in 100 μL PBS, pntranasal injection	1 and 5	[25]
Intratracheal injection	C57BL/6 mice	7 weeks	LLC‐luc and Particulate matter	LLC‐luc cells (2 × 10^6^ cells/mL) were injected intravenously and particulate matter (200 μg) were injected into lung	1, 2, and 10	[26]
	BALB/c mice	—	LLC	3 × 10^5^ cells/mouse, pnjected cell into the trachea	1 and 2	[27]
Heterotopic transplantation						
Subcutaneous graft tumor model	C57BL/6 mice	4–6 weeks	LLC	1 × 10^6^ cells in 200 μL PBS, pnjected cells subcutaneously in the dorsal side of mice	1, 2, and 5	[28]
	C57BL/6 mice	5 weeks	LLC	5 × 10^6^ cells/mouse, pnject cell suspension right armpit of the mice	1, 2, 6, and 11	[29]
	BALB/c nude mice	5–6 weeks	A549	1 × 10^6^ to 1 × 10^7^ cells/mL, s.c.	1, 6, and 11	[30]
Kidney capsule engraftment	Nude mice	3–5 weeks	Cancerous tissues samples of patients with NSCLC	Insert 2–3 tumor pieces (2 × 3 × 3 mm) into the surface of the kidney	1 and 2	[31]
	CD1 athymic mice	6–8 weeks	Tissues silces from primary human lung tumors	Tissue fragments (3 × 3 mm) were grafted under the renal capsule	1	[32]
Metastasis models						
Tail vein injection	SCID mice	12 weeks	A549, NCI‐H3122	1 × 10^5^ A549 cells or 1 × 10^5^ NCI‐H3122 cells suspended in 100 μL of PBS, IV	1 and 2	[33]
	BALB/c nude mice	5 weeks	A549	1 × 10^7^ cells/mouse, IV	1 and 2	[34]
Left ventricular injection	BALB/c nude mice	4 weeks	A549	5 × 10^5^ cells/mouse, left ventriclar injection	1, 2, and 10	[35]
	BALB/c nude mice	4–6 weeks	NCI‐H661	1 × 10^6^ cells were resuspended in 200 μL of PBS, left ventricular injection	1 and 2	[36]
Internal carotid artery injection	SCID mice	5–7 weeks	NCI‐H1915‐luc	1 × 10^5^ cells/mouse, internal carotid artery injection	1 and 2	[37]
	C57BL/6NCrj mice	6 weeks	A549	1 × 10^3^ cells/mouse, internal carotid artery injection	1 and 2	[38]

*Note*: Phenotypes: 1, cell proliferation; 2, tissue invasion and metastasis; 3, oxidative stress; 4, inflammation; 5, immune escape; 6, cell apoptosis; 7, signal path; 8, gene instability and mutation; 9, cell cycle; 10, EMT; 11, immunoinfiltration.

Abbreviations: i.p., intraperitoneal injection; IN, intranasal instillation; ITLI, intratracheal instillation; IV, tail vein injection; s.c., subcutaneous injection.

## 2 ANIMAL MODELS OF CHEMICALLY INDUCED LUNG CANCER

**FIGURE 1:**
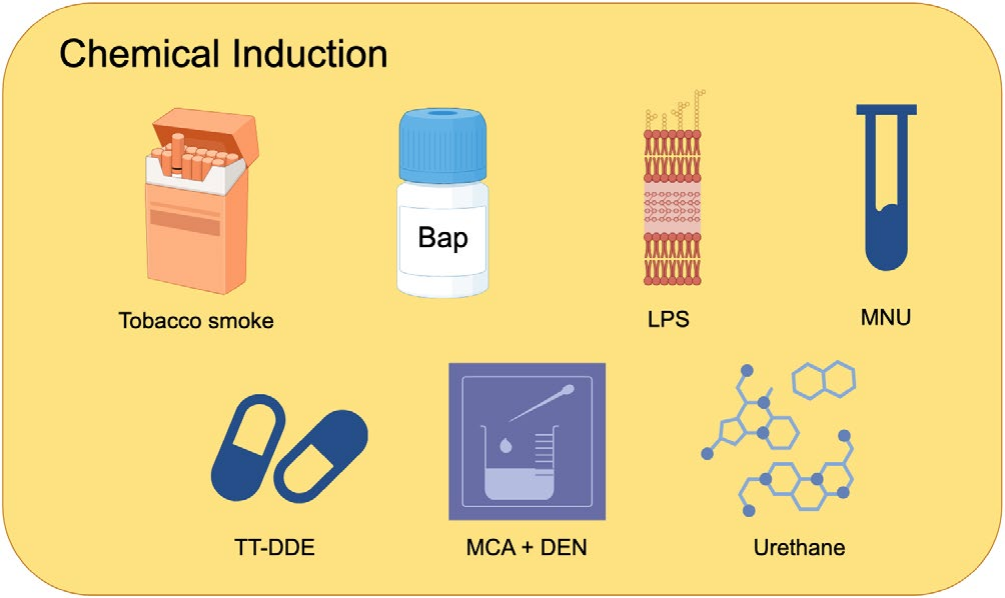
Animal models of chemically induced lung cancer. Bap, benzo[a]pyrene; DEN, diethylnitrosamine; LPS, lipopolysaccharide; MCA, 3‐methylcholanthrene; MNU, *N*‐methyl‐*N*‐nitroxyurea; TT‐DDE, trans‐2,4‐sebacedienal.

Chemically induced lung cancer models represent a critical approach to understanding the disease's pathology by utilizing various agents that mimic the characteristics of lung cancer in animal subjects (Figure 1). Commonly employed chemicals include polycyclic aromatic hydrocarbons (PAHs) and nitrosamines, which are effective in replicating the tumorigenesis associated with lung cancer. In parallel, tobacco smoke models serve as a significant representation of tumor development seen in human smokers, albeit with a potentially prolonged timeline for tumor formation. Within the spectrum of endocrine‐related lung cancers, compounds such as TT‐DDE have been identified as effective in modeling small cell lung cancer and neuroendocrine non‐small cell lung cancer. Urethane also plays a pivotal role, particularly in modeling Kras‐driven lung cancers. Moreover, the contributions of methylation and DNA adduct formation induced by agents like MNU are critical in tumor progression, reflecting various pathological features typical of lung cancers, including adenocarcinoma and squamous cell carcinoma. The interplay between inflammation and lung cancer advancement is further elucidated through the use of agents such as Bap and LPS. Bap primarily induces tumors via mechanisms related to oxidative stress and DNA damage, while LPS often acts in synergy with other carcinogens to elicit immune responses and inflammation that promote tumorigenesis. Lastly, the combination of MCA and DEN in induction models may yield a more nuanced portrayal of the complex tumor microenvironment found in human lung cancer, capturing the intricate nature of tumor development and progression. Wide variety of induction agents enhance our ability to study lung cancer and develop potential therapeutic strategies.

### 2.1 Tobacco smoke induction

Tobacco smoke contains various carcinogens, including benzo[a]pyrene (BaP) and nitroso compounds like 4‐(methylnitrosaminyl)‐1‐(3‐pyridine)‐1‐butanone (NNK). This model simulates the carcinogenic environment of tobacco smoke by either directly exposing animals to the smoke for 2 weeks or injecting them with specific chemicals. BaP is one of the major carcinogenic components. It promotes tumorigenesis by activating intracellular aromatic hydrocarbon receptors (AhR), which in turn triggers inflammation and affects lipid metabolism.^39^ NNK induces lung cancer through several mechanisms, such as DNA methylation^6^, ^7^ and oxidative stress.^8^, ^9^ These processes result in DNA adduct formation, leading to genomic instability in cells.^40^, ^41^ A/J mice and other sensitive strains show a clear carcinogenic response to NNK.^42^, ^43^

#### 2.1.1 Phenotypes

Tobacco smoke often leads to tumors such as adenocarcinoma or squamous cell carcinoma. These tumors show significantly increased proliferation and metastasis potential.^44^ Tobacco smoke can activate immune escape mechanisms through oxidative stress and chronic inflammation. NNK can activate several carcinogenic pathways, including PPARγ /NF‐κB^45^, ^46^ and Wnt /β‐catenin^47^ signaling pathways, which enhance cell proliferation and migration. NNK can inhibit apoptosis^48^ and change the metabolites of intestinal microorganisms, impacting both the immune response and the host's metabolic state.^49^ Changes in the tumor microenvironment, including the release of pro‐inflammatory factors, increased cell proliferation, structural remodeling, and other pathological alterations, have been observed.^50^, ^51^

#### 2.1.2 Applicabilities and limitations

Tobacco smoke‐induced lung cancer models closely mimic human smoking‐related lung cancer. However, this modeling method is time‐consuming, and the exposure duration affects tumor incidence and type. In addition, varying experimental conditions can lead to inconsistent results, limiting the extrapolation of research findings.^52^

### 2.2 Urethane induction

Urethane is a widely used chemical inducer that creates a lung cancer model through intraperitoneal injection in experimental animals, typically at a dose of 1 g/kg, administered three times a week for 12 weeks.^53^, ^54^ Urethane induces lung cancer primarily by inhibiting DNA repair mechanisms^10^, ^11^ and promoting apoptosis.^12^, ^55^ Furthermore, its metabolites bind to intracellular biomacromolecules, which contributes to cell dysfunction and genomic instability.^56^

#### 2.2.1 Phenotypes

Lung cancer cells in this model often show abnormal shapes and changes in their nuclei, as well as signs of growth. Studies^57^, ^58^ have noted mutations in genes related to tumors, such as p53 and Kras. These mutations are somewhat similar to those found in human lung cancer, as noted in references.^59^

#### 2.2.2 Applicabilities and limitations

This model is easy to operate and commonly linked to mutations in the Kras gene, making it a useful tool for simulating KRAS‐driven lung cancer. The ethyl carbamate‐induced lung cancer models may exhibit resistance to certain chemotherapy drugs. This characteristic provides a valuable experimental basis for investigating the mechanisms of drug resistance in lung cancer.^60^, ^61^ Nonetheless, the varying sensitivities of different animal strains to ethyl carbamate may influence the model's consistency and reproducibility.

### 2.3 Bap induction

BaP is a polycyclic aromatic hydrocarbon commonly found in the environment, particularly in tobacco smoke and automobile exhaust. In this model, researchers exposed animals to BaP either through intraperitoneal injection (1 mg once a week for 4 weeks) or by adding it to their drinking water (50 mg/kg, two times a week for 4 weeks).^62^ BaP can cause mutations and tumors. It does this by triggering intracellular oxidative stress and leading to the formation of DNA adducts.^63^, ^64^

#### 2.3.1 Phenotypes

Bap is involved in cell cycle regulation, particularly in the G1 and G2/M phases, where it can inhibit apoptosis and promote cell proliferation, accelerating tumor progression.^13^, ^14^, ^65^ Exposure to Bap typically impacts the function and structure of various organs, including the liver and lungs, resulting in pathological features such as inflammation, fibrosis, and tumor formation.^66^ Bap also alters the body's immune response, affecting both the immune system and the tumor microenvironment, which promotes tumor development.^67^, ^68^ Lung cancer cells induced by benzopyrene frequently exhibit increased proliferation, reduced apoptosis, and enhanced metastatic potential.^69^, ^70^, ^71^

#### 2.3.2 Applicabilities and limitations

The BAP‐induced lung cancer model is user‐friendly, allows for effective dose and timing control, and is well‐suited for large‐scale screening tests. The inflammatory response activated in lung tissue after benzopyrene exposure is closely linked to tumorigenesis, underscoring its significant role in lung cancer development.^72^

### 2.4 *N*‐methyl‐*N*‐nitroxyurea (MNU) induction

MNU is a chemical that attaches a methyl group to DNA, resulting in the formation of DNA adducts. This process causes gene mutations and can lead to tumor formation.^15^ In this model, lung cancer is primarily induced by either intraperitoneal (50 mg/kg for 20–26 weeks) or intravenous injection of MNU solvent, allowing control over the timing of tumor formation through dose and injection frequency. The incidence of lung cancer increases significantly at doses of 50 mg/kg or more.^73^

#### 2.4.1 Phenotypes

MNU causes lung cancer by inducing Kras mutations. It also increases the tumor mutation burden, enhances the immune response, and may affect the effectiveness of immune checkpoint inhibitors.^74^, ^75^

#### 2.4.2 Applicabilities and limitations

The MNU‐induced lung cancer model has a high tumor incidence and can simulate multiple pathological features of human lung cancer, including adenocarcinoma and squamous cell carcinoma.^16^ MNU treatment in FVB‐Trp53 heterozygote mice effectively induced lung tumor development, with genetic characteristics resembling those of human lung cancer.^73^

### 2.5 Trans‐2,4‐sebacedienal (TT‐DDE) induction

TT‐DDE, often present in cooking smoke, is recognized for its tendency to accumulate in living organisms and its ability to disrupt hormonal functions.^76^ This substance can induce lung cancer when administered via intratracheal instillation (24 mg/kg for 8 weeks).^77^ Additionally, TT‐DDE can enhance inflammation and trigger cancer‐related signaling pathways inside cells.^17^, ^78^

#### 2.5.1 Phenotypes

The lung cancer model induced by TT‐DDE primarily demonstrates its effects on the endocrine and immune systems. In vivo, TT‐DDE metabolites can disrupt the endocrine system, cause an imbalance in cell proliferation and apoptosis,^78^, ^79^ elevate oxidative stress markers in lung tissue, suppress immunity, and ultimately result in cell damage and gene mutations.^77^, ^80^ Mice that are exposed to prolonged TT‐DDE show an increase in alveolar macrophages, epithelial overgrowth, and the formation of granulomatous nodules, which may also aid in the growth and spread of tumor cells by altering JNK signaling pathways.^81^

#### 2.5.2 Applicabilities and limitations

The TT‐DDE model is effective for evaluating the efficacy of anti‐tumor drugs, especially those targeting endocrine‐related tumors. Long‐term exposure to TT‐DDE should be studied further, as most existing research only examines short‐term effects. Because TT‐DDE is bioaccumulative, individual differences among animals may affect the experimental results.

### 2.6 3‐Methylcholanthrene (MCA) combined with diethylnitrosamine (DEN) induction

The combined induction model of MCA and DEN for lung cancer simulates the effects of multiple carcinogens. This model induces lung cancer by instilling a suspension of iodized oil containing MCA and DEN into the tracheas of animals.^18^, ^19^ Specifically, 10 mg of MCA and 10 μL of DEN are instilled into the left lobe on days 15, 35, 55, 65, and 75. MCA is a known carcinogen that forms DNA adducts, disrupting key regulatory genes involved in cell cycle regulation and apoptosis. When paired with promoters like butylated hydroxytoluene (BHT), it significantly increases the risk of tumorigenicity.^20^ DEN causes tumor formation through its metabolites, which damage DNA and promote oxidative stress in lung tissue.^82^, ^83^ The combination of these two chemicals significantly enhances DNA damage and inhibits cell repair, increasing the frequency of tumor occurrence.

#### 2.6.1 Phenotypes

This model represents the process of changing normal bronchial tissue into squamous tissue, which is then followed by abnormal cell growth, localized cancer, and invasive cancer.^18^ Throughout this process, immune cell infiltration and the expression of immune factors are observed. Additionally, the positive expression rates of p27, p57, and other proteins increase as cancer progresses.^20^ The combination of MCA and DEN induces significant lung inflammation and promotes neutrophil recruitment, which is essential for tumor development.^82^

#### 2.6.2 Applicabilities and limitations

When MCA is combined with DEN, it significantly increases lung cancer incidence and provides a better representation of the multifactorial carcinogenic mechanisms in humans. The tumor occurrence in the combined treatment group was significantly higher than in the single treatment group, indicating that the synergistic effect of chemical carcinogens plays a crucial role in lung cancer development.^83^ However, the modeling cycle is lengthy,^84^ and there is a high reliance on animal models.

### 2.7 Lipopolysaccharide (LPS) induction

LPS, which is a component of the outer membrane found in gram‐negative bacteria, acts as an effective inflammatory agent.^85^ The common modeling methods used to induce a chronic inflammatory response have included intraperitoneal injection,^16^, ^86^ caudal vein injection,^21^, ^87^ and tracheal injection or infusion,^21^, ^22^ which in turn have promoted tumor occurrence. In experiments, LPS was often combined with NNK, typically at a dosage of 3 mg of NNK and 5 μg of LPS for a duration of 10 weeks. Tumor proliferation and immune escape were then observed.^21^

#### 2.7.1 Phenotypes

LPS‐mediated chronic inflammatory environments contain proinflammatory factors that can cause genomic instability in cells, increasing the likelihood of mutations.^22^ Inflammatory cell infiltration and activation release various growth factors and cytokines that promote tumor cell growth and metastasis. Inflammation also affects the tumor microenvironment and modifies immune cell function, facilitating tumor evasion and progression.^16^, ^88^ Chronic inflammation mediated by LPS can induce T cell failure by regulating the PD‐1/PD‐L1 immune checkpoint axis, impairing the immune system's ability to recognize and eliminate tumor cells.^89^

#### 2.7.2 Applicabilities and limitations

LPS effectively simulates the impact of chronic inflammation on tumorigenesis, serving as a valuable experimental platform for studying inflammation‐related lung cancer. However, differences may exist between the inflammatory response induced by LPS and the pathogenesis of human lung cancer. Therefore, the biological relevance of this model requires further verification.^90^ Furthermore, how LPS is administered, including the method and dosage, may impact the reproducibility of the experimental results. Moreover, the LPS‐induced model primarily investigates the relationship between inflammation and tumorigenesis, which may overshadow the impact of other environmental factors, like smoking and pollution, on lung cancer.

## 3 LUNG CANCER TRANSPLANTATION MODELS

There are three primary methods for creating animal models of lung cancer transplantation: orthotopic transplantation, heterotopic transplantation, and metastasis models. These models originate from three primary sources: grafts derived from patient tissues, grafts from cell lines, and grafts from circulating blood tumor cells. Each of these models, derived from different sources, can address a wide range of research needs.

### 3.1 Orthotopic transplantation of lung cancer animal models

**FIGURE 2:**
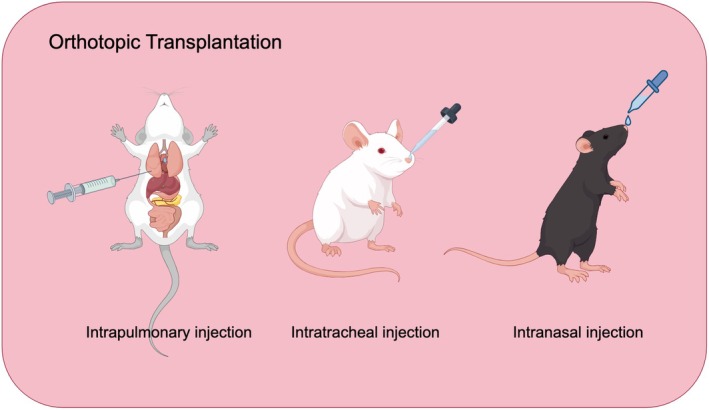
Orthotopic transplantation of lung cancer animal models.

Lung cancer modeling methods are crucial for understanding tumor growth and development, and they typically encompass intrapulmonary, intranasal, and intratracheal injection techniques (Figure 2). Intrapulmonary injection involves administering lung cancer cell lines or chemicals directly into the lungs, allowing for better control over tumor localization and growth rates. The endotracheal injection model, on the other hand, entails injecting carcinogens directly into the trachea, thereby significantly influencing the physiological state of the lungs, which is advantageous for accurately reflecting the metastasis and diffusion characteristics of tumor cells within the airways. However, it presents complexities in replicating the tumor microenvironment, which must be accurately replicated in order to investigate mechanisms of tumor immune escape. Furthermore, the intranasal injection model serves to simulate tumor spread in the upper respiratory tract and its effects on adjacent tissues via nasal administration. That is vital for elucidating the early pathogenesis of lung cancer and developing effective prevention strategies. Additionally, the intranasal injection model opens new avenues for researching inhaled drug delivery in relation to tumors. Overall, these modeling methods are integral to advancing our understanding of lung cancer dynamics and therapeutic strategies.

#### 3.1.1 Intrapulmonary injection

In this model, researchers inject lung cancer cells into the lung parenchyma of experimental animals.^91^ Alternatively, they use a direct percutaneous injection^23^, ^24^ after surgically separating the tissue to allow for in situ growth. Subsequently, after the injection of luciferase‐labeled cells, researchers can monitor tumor growth and metastasis in real time using in vivo imaging.

##### Phenotypes

Intrapulmonary injection‐induced lung cancer models typically exhibit rapid tumor growth and are highly aggressive.^92^ The levels of biomarkers such as IL‐6 and TNF‐α are significantly elevated and strongly associated with the inflammatory microenvironment of tumors.^91^

##### Applicabilities and limitations

The intrapulmonary injection model effectively assesses how various treatment regimens influence the tumor microenvironment, particularly in immunotherapy and targeted therapy studies. Additionally, this model can be used to study how the tumor microenvironment affects tumor growth, including the interactions between tumor cells and host cells, as well as the role of tumor‐associated fibroblasts, which is crucial for understanding tumor aggressiveness and metastasis.^93^, ^94^ However, this modeling method is more complex to perform. Unlike subcutaneous tumors, which can be directly observed for growth, tumors grow within the lung in this model. Injecting tumor cells into the lung can lead to tumor spread and metastasis to the pleura, resulting in multifocal growth. This complicates the study of individual tumors^95^ and places high technical demands on surgeons.

#### 3.1.2 Intranasal injection

In this model, lung cancer cells, such as Lewis Lung Carcinoma (LLC) cells (1 × 10^5^ to 1 × 10^7^ cells in 100 μL PBS) or NCI‐H460 cells (1 × 10^5^ cells in 100 μL PBS), are injected intranasally into mouse lungs to establish a lung cancer model that effectively simulates the natural progression of cancer in vivo, with tumor proliferation evident in both the left and right lungs.^25^

##### Phenotypes

The model effectively simulates key pathological features of human lung cancer, such as tumor growth, spread, and changes in the microenvironment. Histological analysis revealed that the tumor cells appeared abnormal, while the surrounding tissues exhibited a marked inflammatory response, indicating changes in the tumor microenvironment.^96^

##### Applicabilities and limitations

Compared to direct pulmonary injection, intranasal injection better mimics the natural progression of cancer in the body, leading to a more complete pathological formation of lung cancer. Consequently, this model can evaluate the effectiveness of anticancer drugs more accurately.^97^, ^98^ However, although the procedure is relatively simple, individual differences in the nasal cavity and lung structure of mice may affect the model's consistency and repeatability. Additionally, intranasal injection has limitations compared to the orthotopic transplantation model when evaluating the tumor microenvironment and local aggressiveness.^99^

#### 3.1.3 Intratracheal injection

In this model, a syringe is inserted into the trachea through a bronchofiberscope or tracheal intubation. Tumor cell suspensions, specifically Lewis Lung Carcinoma‐luciferase (LLC‐luc) containing 3 × 10^5^ cells in 200 μL PBS, were injected into the tail vein. In addition, 200 μg of particulate matter was administered to induce lung cancer.^26^, ^27^

##### Phenotypes

After the endotracheal injection, tumor cells quickly multiply in the lung tissue, resulting in distinct tumor nodules characteristic of either lung adenocarcinoma or squamous cell carcinoma.^100^ Following the injection, the physiological indicators of the animals—including body weight, mobility, and respiratory function—may show changes, reflecting physiological stress.^99^ Furthermore, the establishment of the model is accompanied by inflammatory reactions, particularly in the tissues surrounding the tumor, where a high density of immune cells is observed.^101^, ^102^, ^103^

##### Applicabilities and limitations

This model better simulates the natural process of cancer progression in the body, with tumor proliferation clearly visible in the lungs. While intratracheal injection offers advantages, it has a significant limitation due to its high technical requirements, making the procedure challenging. Intratracheal injection results in lower tumor formation and metastasis rates compared to other cancer models.^104^, ^105^

### 3.2 Heterotopic transplantation of lung cancer animal models

**FIGURE 3:**
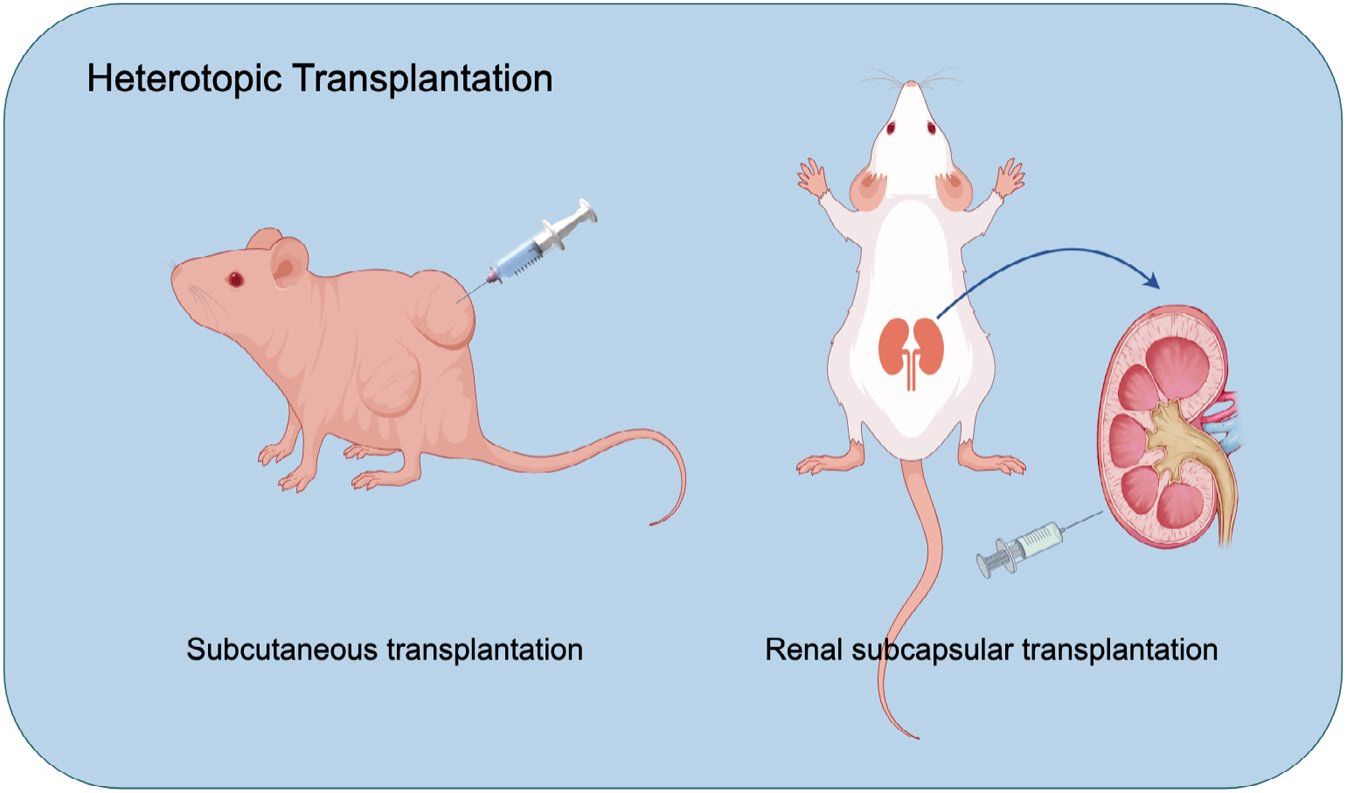
Heterotopic transplantation of lung cancer animal models.

The heterotopic lung cancer model serves as a valuable platform for investigating tumor dynamics by implanting lung cancer cells or tumor tissues into non‐pulmonary sites, like subcutaneous or subcapsular kidney regions (Figure 3). Among these, subcutaneous graft models are particularly notable for exhibiting accelerated tumor growth and improved survival rates. This is attributed to the subcutaneous tissue's abundant blood supply and less immunosuppressive nature, which facilitate the proliferation and spread of the implanted cancerous tissues. Conversely, while the renal subcapsular transplantation model offers a supportive environment for tumor development, its anatomical closeness to the kidney may invoke heightened immune surveillance. This can complicate tumor growth and metastasis due to the increased immune response, leading to slower progression compared to subcutaneous models. Overall, these modeling methods are integral to advancing our understanding of lung cancer dynamics and therapeutic strategies.

#### 3.2.1 Subcutaneous transplantation of lung cancer animal models

In this model, fresh samples or lung cancer cells from patients were implanted directly under the skin of animals. Commonly used cell lines include the Lewis and A549 cell lines. The inoculation sites included the armpit,^28^, ^29^ back,^106^, ^107^ hip,^30^ foot pad,^108^ and others. To accurately reflect the tumor's growth and metastasis characteristics, researchers often vary injection doses and cell sources.^109^ Additionally, various immunodeficient mouse strains, such as nude, NOD/SCID, and transgenic mice, effectively accept human lung cancer cell lines and simulate the disease's biological characteristics.^110^

##### Phenotypes

Subcutaneously transplanted tumor cells can form large tumor masses in mice and exhibit a notable growth rate over time. The tumor may metastasize to other organs. Pathologically, the tumor tissue is characterized by high cell atypia and increased nuclear division, which indicate its malignancy.^111^, ^112^

##### Applicabilities and limitations

Subcutaneous transplantation of lung cancer tumor models offers several advantages, including ease of operation, low cost, rapid tumor model establishment, and the ability to directly observe tumor growth and drug responses, providing a solid platform for new drug development and efficacy evaluation. However, the inoculation occurs beneath the skin, which lacks the lung's microenvironment and blood supply, leading to potential differences in the tumor's biological characteristics compared to primary lung cancer tumors.^113^, ^114^ Furthermore, while the use of immunodeficient mice helps eliminate interference from immune responses, it also restricts the model's ability to accurately replicate the immune microenvironment of human tumors. Lastly, the metastatic properties of subcutaneous tumors may be less pronounced than those of in situ tumors, limiting their applicability in metastasis studies.

#### 3.2.2 Renal subcapsular transplantation of lung cancer animal models

To simulate tumor growth and metastasis, researchers transplant lung cancer tissue samples from patients with non‐small cell lung cancer (NSCLC), measuring 2 × 3 × 3 mm, or cells from beneath the kidney capsule of experimental animals.^31^, ^32^

##### Phenotypes

In a model of lung cancer transplanted under the renal capsule, tumor growth is rapid and aggressive. A few weeks after implantation, the tumor expands rapidly and may metastasize to other organs, including the lungs, liver, and bones, via the blood or lymphatic system.^115^ The ability of tumor cells to metastasize is closely linked to their aggressiveness, the remodeling of the extracellular matrix, and alterations in the tumor microenvironment. Tumor cells secrete cytokines that modify the function of nearby immune cells, which in turn inhibits the anti‐tumor immune response. Additionally, the acidic and hypoxic conditions of the tumor microenvironment can impair immune cell activity, thereby promoting tumor progression and metastasis.^116^

##### Applicabilities and limitations

This model typically requires surgical intervention. The subcapsular transplantation model has a higher tumor formation rate than subcutaneous transplantation and more accurately reflects the tumor's biological characteristics and therapeutic response. The subcapsular renal transplantation model has limitations due to its procedural complexity and potential effects on the animal's survival and health post‐surgery. Additionally, the kidney's physiological functions may restrict tumor cell growth due to local blood flow and oxygen supply, leading to different growth characteristics compared to tumors in other body parts.^117^

### 3.3 Animal models of lung cancer metastasis

**FIGURE 4:**
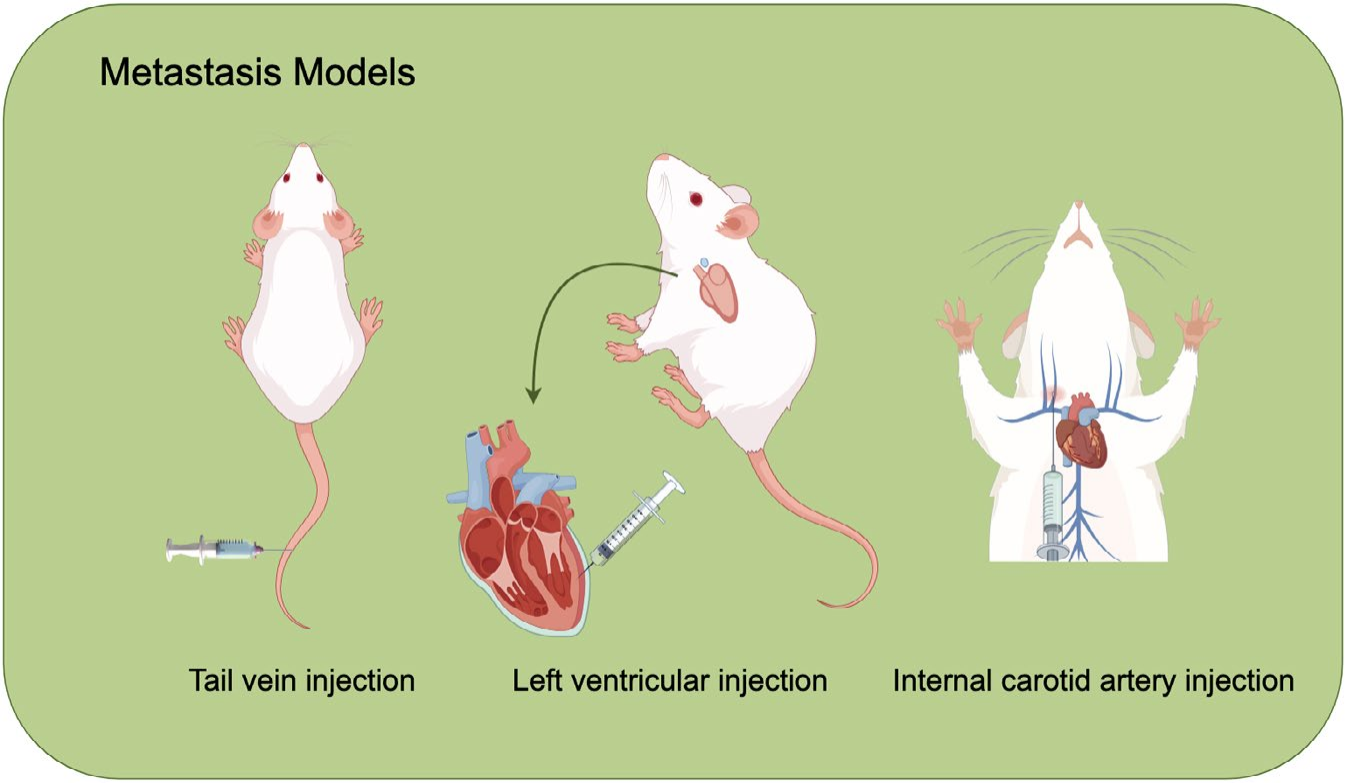
Metastasis models of lung cancer.

Lung cancer metastasis significantly exacerbates patients' conditions, leading to decreased therapeutic effectiveness and survival rates, making it a major contributor to poor prognoses. Various models have been developed to simulate the middle and late stages of lung cancer metastasis, utilizing fluorescent‐labeled lung cancer cell lines and different injection methods, including tail vein, left ventricular, and internal carotid artery injections (Figure 4). The caudal vein model is noted for increasing metastasis rates, particularly in common sites such as the liver and bones. This heightened rate may be attributed to the direct connection between the tail vein and the liver, facilitating rapid entry of cancer cells into liver circulation and thereby increasing the likelihood of liver metastasis. Conversely, left ventricular injection tends to induce metastasis primarily in the lungs and heart, although its overall effectiveness is relatively low due to the dilution of cancer cells by the widespread blood flow from the heart before they can reach target organs. In addition, internal carotid artery injection has been associated with a specific increase in brain metastasis, aligning with the blood flow path that directly supplies the brain. Metastatic tumor formation occurs as tumor cells disseminate via the blood and lymphatic systems to various body parts. Common critical organs affected include lymph nodes, bones, brain, and liver, underscoring the importance of selecting appropriate models for studying lung cancer metastasis. While the caudal vein model demonstrates high rates of metastasis, it is essential to evaluate its impact on patient survival in conjunction with the specific metastatic sites and therapeutic approaches employed.

#### 3.3.1 Lung cancer metastasis models induced by tail vein injection

This model simulates lung cancer metastasis by injecting lung cancer cells into the tail vein of mice. Researchers can then observe the spread of these cells in the lungs and other distant organs through blood circulation. This provides an excellent platform for studying the mechanisms of lung cancer blood metastasis.^33^, ^34^ Surveillance typically involves a combination of imaging techniques, such as CT and MRI, along with histological analysis. It may also include detecting specific markers in blood or tissue to assess lung cancer progression and treatment effectiveness.^104^, ^118^

##### Phenotypes

Tumor cell metastasis is closely linked to surface markers,^119^ cell–cell interactions,^120^, ^121^ and microenvironmental factors.^122^ The histological features of metastatic lung cancer are characterized by tumor cell heterogeneity, invasion, and angiogenesis.^123^, ^124^ Immune and stromal cells within the tumor microenvironment play a crucial role in regulating metastasis. Pathological studies indicate that the histological features of metastatic lung cancer often include tumor cell heterogeneity, invasion, and angiogenesis.

##### Applicabilities and limitations

The tail vein injection model of lung cancer metastasis effectively simulates how tumor cells spread from the primary site to the lungs via the bloodstream. This process is closely linked to the type and quantity of tumor cells, as well as the genetic background of the mice.^125^, ^126^ Additionally, tail vein injection is easy to perform and highly repeatable. Compared to other invasive modeling methods, tail vein injection exerts less physiological stress on mice, making it suitable for large‐scale drug testing and screening. However, this model has specific limitations. Variations in the biological characteristics of different cell lines can affect the model's repeatability and accuracy.

#### 3.3.2 Lung cancer metastasis models induced by left ventricular injection

This model is more complex for inducing lung cancer metastasis and more accurately simulates how tumor cells enter the systemic circulation through the heart. Additionally, the metastasis rate increases with the number of injected cells.^127^

##### Phenotypes

Metastasis involves several complex physiological mechanisms, including heart pumping capacity, hemodynamics, and the interaction between tumor cells and vascular endothelial cells. Once in the bloodstream, tumor cells can colonize and grow in target organs by adhering to and penetrating endothelial cells. Tumor cells can increase their metastatic ability in the bloodstream through epithelial‐mesenchymal transition (EMT) and degradation of the extracellular matrix.^35^ The high‐pressure environment of the left ventricle may affect cell survival and metastasis, while the heart's systolic function and blood flow rate play key regulatory roles in tumor cell metastasis.^127^ Pathologically, metastases typically exhibit adenocarcinoma structures along with significant necrosis and inflammatory responses. The expression levels of common markers such as Ki‐67 and CD31 are significantly increased in immunohistochemical staining.^36^

##### Applicabilities and limitations

This model can investigate how lung cancer metastasizes and the heart's role in this process. The metastasis patterns observed in lung cancer patients closely match those seen in experimental animal models, indicating that this model has significant practical applications in both basic research and the clinical management of lung cancer.^128^, ^129^ However, the cardiac microenvironment may influence the growth and metastatic behavior of tumor cells.^130^

#### 3.3.3 Lung cancer metastasis models induced by internal carotid artery injection

In this model, ultrasound or X‐ray guidance was employed to locate the target artery accurately. A catheter was then inserted through a small incision, or a micro‐syringe was used to inject a suspension of lung cancer cells. Regular imaging examinations, including small animal imaging, CT, and MRI, were performed after surgery to monitor the tumor's growth and metastasis.^37^

##### Phenotypes

The ability of tumor cells to survive in the bloodstream, interact with vascular endothelial cells, and adapt to their microenvironment are crucial factors that influence metastasis success. Tumor cells increase their metastatic potential by secreting specific cytokines, including transforming growth factor β (TGF‐β) and matrix metalloproteinases (MMPs), which promote both angiogenesis and cell migration.^127^ Additionally, the unique traits of tumor cells significantly influence the metastasis process, with stem‐like cells demonstrating greater survival and metastatic potential.^35^ Pathologically, metastases are characterized by tumor cell heterogeneity and the remodeling of the microenvironment. These changes are often accompanied by inflammatory responses and immunosuppression, which create new targets for the early diagnosis and treatment of lung cancer.^128^

##### Applicabilities and limitations

Internal carotid artery injection can be employed to induce lung cancer metastasis for studying metastasis to the brain.^38^ The internal carotid artery injection mimics the actual process of cancer cell accumulation, adhesion, invasion, and penetration through the blood–brain barrier, leading to metastasis in the brain's terminal capillary bed. This approach allows for a more realistic investigation of lung cancer's blood‐transport metastasis mechanism.^131^ However, the procedure demands a high level of skill from the operator, and there is a risk of inducing non‐specific metastasis.

#### 3.3.4 Other lung cancer metastasis models

Lymphatic and bone metastasis are among the most common and severe complications of lung cancer. Lymphatic metastasis can occur by injecting lung cancer cell lines either into the bloodstream or directly into lung. Tumor cells enhance their metastatic potential by secreting specific growth factors like VEGF‐C, which promote the formation of lymphatic vessels.^132^ Immune and stromal cells in the tumor microenvironment also play a crucial role in metastasis. Some immune cells can promote the migration and invasion of tumor cells, resulting in the occurrence of lymphatic metastasis. Lung cancer with lymphatic metastasis typically shows pathological features such as lymph node enlargement and invasion of lymphatic vessels. These features can be confirmed through imaging and histological examinations.^133^

The lung cancer bone metastasis model is created by injecting a lung cancer cell suspension either through the tail vein or directly into the bone marrow cavity. Imaging technologies like PET‐CT are then used to monitor the progression of metastasis and bone injury in real time, allowing for the evaluation of the model's effectiveness. The intravenous injection method has a higher success rate than direct injection, with a metastasis rate exceeding 80%. A successful bone metastasis model should accurately reflect the growth and metastatic characteristics of tumor cells in bone tissue. The evaluation criteria mainly include the formation of metastases, the change of tumor load, the degree of bone injury, and the survival time of mice. Lung cancer cells can release a variety of growth factors in bone tissue, such as transforming growth factor β (TGF‐β) and bone morphogenetic protein (BMP), which promote the occurrence of bone metastasis. Biologically, lung cancer bone metastases often consist of more aggressive and drug‐resistant cells that can adapt to and survive in the bone microenvironment. Pathologically, lung cancer bone metastases typically result in bone destruction and an inflammatory response in the bone marrow, which can lead to pain and other clinical symptoms. In addition, lung cancer bone metastasis may also affect bone metabolism, leading to complications such as osteoporosis.

### 3.4 Lung metastasis models of tumor

Various tumor types, such as melanoma, kidney cancer, breast cancer, and liver cancer, frequently metastasize to the lungs in clinical settings. Lung metastasis models for tumors include tail vein injection, in situ inoculation, genetically engineered mice, and exosome induction. Among these, melanoma and liver cancer lung metastasis models are mainly created using tail vein injection, a straightforward method with a high metastasis rate that is suitable for large‐scale experiments. However, the method fails to accurately mimic the natural metastasis process. Orthotopic transplantation is the primary method for constructing lung metastasis models of soft tissue sarcoma, kidney cancer, and breast cancer. This approach better simulates the natural metastasis process and is ideal for studying metastasis mechanisms and drug screening. However, this method is technically challenging, requiring high skill levels and extended observation time.

#### 3.4.1 Melanoma lung metastasis models

Establishing a melanoma lung metastasis model typically involves tail vein injection or subcutaneous implantation. Tail vein injection directly introduces melanoma cells into the bloodstream, simulating the tumor cell metastasis process. The injection of 5 × 10^5^ B16‐F10 cells in 200 μL of PBS into the tail vein effectively establishes a lung metastasis model, with observable lung metastasis appearing after 2 weeks.^134^ Once the model is established, imaging and histological analysis are used to measure the number and size of lung metastases, allowing for an assessment of tumor growth and metastasis.

##### Phenotypes

After injection, melanoma cells enter the pulmonary microvessels via the bloodstream. By the second week, a few metastases appeared and increased rapidly. By the fourth week, some mice had lung surfaces covered with metastases.^135^ Histological analysis using H&E and Bouin's fixed staining revealed that the lung metastases exhibited a typical focal or clumpy distribution. Over time, new metastases continued to emerge, leading to a significant increase in tumor volume. In lung metastases, melanoma cells showed high proliferative activity, and the positive rate of Ki67 was also high, indicating that tumor cells have strong proliferation and invasion ability.^136^ Tumor cell metastasis alters the lung microenvironment, mainly through the recruitment and polarization of tumor‐associated macrophages (TAMs). These macrophages enhance the aggressiveness and metastasis of melanoma cells by secreting cancer‐promoting factors and cytokines.^120^ Melanoma cells interact with surrounding cells through exosomes to regulate the tumor microenvironment and promote the occurrence of metastasis.^137^, ^138^

##### Applicabilities and limitations

This model effectively simulates how melanoma cells colonize and spread within pulmonary microvessels. The metastases formed in this model closely resemble clinical melanoma lung metastases, characterized by a focus‐like or clumpy distribution. This similarity provides a crucial foundation for further investigation into the mechanisms of metastasis.

#### 3.4.2 Renal carcinoma lung metastasis models

Common methods to create lung metastasis models of renal cancer include tail vein injection, orthotopic transplantation, and genetically engineered mouse models. Human renal cancer cells were either injected into the tail vein at a concentration of 5 × 10^5^ cells in 100 μL of PBS or injected into the subcapsular space of the kidneys at a concentration of 5 × 10^4^ cells per mouse in either nude or immunocompromised mice.^139^ Frequently utilized renal cancer cell lines include SN12‐PM6 and 786‐O. Genetically engineered models employing CRISPR/Cas9 technology to modify genes associated with kidney cancer metastasis can induce the disease and its spread in mice. This approach more accurately mimics the process of kidney cancer cell metastasis to the lungs.^140^ Extracellular vesicles from renal carcinoma cells significantly contribute to the formation of pre‐metastatic niches.^141^ Consequently, researchers created a new metastasis model by extracting and analyzing the composition of extracellular vesicles secreted by kidney cancer cells. These vesicles were then injected into mice to observe their effects on the lung microenvironment.

##### Phenotypes

Renal cancer cells exhibit a strong tendency to metastasize to the lungs. They also create a pulmonary immunosuppressive microenvironment by secreting complement protein C3 in extracellular vesicles (EVs) and attracting tumor‐associated macrophages (TAMs) and polymorphonuclear myeloid immunosuppressor cells (PMN‐MDSCs), further facilitating metastasis.^142^ There are sex differences in the lung metastasis of renal carcinoma, with high expression of the androgen receptor (AR) closely linked to this process. In nude mouse models, increased AR expression significantly enhances lung metastasis of renal cancer.^143^ The expression of the LAPTM5 gene in renal cancer cells is crucial for lung metastasis. LAPTM5 maintains the stem cell characteristics of renal cancer cells by inhibiting the function of pulmonary bone morphogenetic protein (BMP), thereby promoting lung‐specific metastasis.^144^

##### Applicabilities and limitations

The animal model for lung metastasis in renal cancer effectively simulates the clinical lung metastasis process and exhibits a high rate of tumor formation and metastasis. However, many models rely on immunodeficient mice, such as nude mice, which cannot fully replicate the human immune response due to their incomplete immune systems. Additionally, genetically modified mice are unsuitable for large‐scale experiments because of high costs and technical challenges.

#### 3.4.3 Breast cancer lung metastasis models

Breast cancer lung metastasis models include the spontaneous lung metastasis model, the tail vein injection model, the exosome induction model, and genetically engineered mice. Commonly used cell lines, such as MDA‐MB‐231 and 4 T1, use a viral vector system to select cells that stably express green fluorescent protein (GFP). 2 × 10^5^ Luciferase‐labeled MDA‐MB‐231 cells were injected into mice through the caudal vein.^145^ The spontaneous metastasis model mimics natural tumor growth and distant metastasis by inoculating breast cancer cells either into the mammary fat pad or subcutaneously in immunodeficient mice.^146^ In the exosome‐induced model, 4 T1 tumor‐bearing mice, which have 5 × 10^5^ cells seeded into breast pads, are placed in a chronic stress environment to stimulate the secretion of exosomes rich in proteins such as SP1, promoting lung metastasis of breast cancer.^147^

##### Phenotypes

In the lung metastasis model of breast cancer, tumor cells migrate to the lungs via the bloodstream, forming tiny metastases. Over time, these metastases increase in size and eventually form distinct pulmonary nodules. Chronic stress significantly promotes lung metastasis of breast cancer through exosomes (TDEs) secreted by tumor cells. These exosomes induce immunosuppressive microenvironments by activating the TLR4‐NFκB‐IL‐1β pathway in pulmonary neutrophils.^148^ In the 4 T1 breast cancer model, the number and volume of lung metastases increased with time, and the size distribution of metastases showed dynamic changes.^139^, This change can be quantitatively described by mathematical models. During metastasis, the pulmonary immune microenvironment undergoes significant changes, characterized by an increase in neutrophils and monocytes and a decrease in alveolar macrophages.^149^

##### Applicabilities and limitations

The lung metastasis animal model for breast cancer effectively simulates how breast cancer cells spread to the lungs via the bloodstream, aiding the study of metastatic mechanisms. Researchers can successfully create lung metastasis models of 4 T1 breast cancer cell lines using either tail vein injection or subcutaneous injection. This model can study the formation of pre‐metastatic niches and analyze dynamic changes in the migratory microenvironment. Chronic stress changes the composition of exosomes released by tumor cells, thereby fostering the development of a pulmonary immunosuppressive microenvironment. However, investigating the role of exosomes in metastasis necessitates advanced experimental equipment and techniques, such as isolating and identifying exosome components and analyzing their effects on immune cells.

#### 3.4.4 Liver cancer lung metastasis models

Common methods to create lung metastasis models for hepatocellular carcinoma include in situ injection, tail‐vein injection, exosome induction, and genetically engineered mouse models. Commonly used cell lines are HepG2, H22, and others. Hep3B and Huh‐7 cells, consisting of 2 × 10^6^ cells in 50 μL of serum‐free DMEM, were cultured in vitro and then injected into the left liver lobe.^150^ Genetic engineering techniques can be employed to investigate the roles of SENP3 and other specific genes in the progression and metastasis of liver cancer.^151^

##### Phenotypes

In a liver cancer lung metastasis model, liver cancer cells circulate through the bloodstream to the lungs, forming small metastases. After injecting H22 liver cancer cells into the tail vein, small metastases appeared in the lungs of mice within 7 days. The number of metastases gradually increased at 14 and 21 days.^152^ The increase of matrix stiffness regulates the formation of pre‐metastatic lung niche through miRNAs such as let‐7d‐5p and miR‐365a‐5p in exosomes.^153^ These exosomes enhance glucose metabolism by targeting metabolic pathways in lung fibroblasts, creating favorable conditions for tumor cell colonization and growth. Liver cancer cells recruit tumor‐associated macrophages (TAMs) by secreting specific factors, such as CCL20, which reshapes the pulmonary immune microenvironment and suppresses the anti‐tumor immune response.^154^ A large number of immune cells infiltrated the metastatic site, including TAMs, CD8+ T cells and NK cells. The expression of SENP3 and other genes regulates the infiltration of these immune cells, thereby influencing tumor progression.^151^ Liver cancer cells showed strong proliferation and invasion ability after colonization in the lung, and immunohistochemical results showed positive expression of AFP and GPC‐3 markers in metastatic lesions.^155^ The miR‐7/TGF‐β2 axis was found to be closely related to lung cancer metastasis induced by acidic tumor microenvironment, suggesting that liver cancer cells may depend on acidic changes in the microenvironment during metastasis.^156^

##### Applicabilities and limitations

Caudal vein injection can effectively create a liver cancer lung metastasis model that has a high tumor formation rate and is easy to perform, In the high‐concentration cell injection group (2 × 10^5^ cells in 200 μL PBS), the lung tumor formation rate reached 70% within 14 days after the injection.^152^ This model can also be utilized to study the formation of the pre‐metastatic niche, the dynamic changes in the metastatic microenvironment, and to evaluate the therapeutic effects of anti‐metastatic drugs. However, in genetically engineered mouse models, the DNA mutation profile may differ from that of human liver cancer, which could compromise the validity of the findings for clinical use.

#### 3.4.5 Other models of lung metastasis from tumors

Besides the cancers mentioned earlier, colorectal cancer, thyroid cancer, and head and neck squamous cell carcinoma frequently metastasize to the lungs. When colorectal cancer cells are injected into the tail vein, they first travel to the liver via the portal vein and then to the lungs through systemic circulation. Lung metastases typically present as multiple nodules and are often associated with significantly elevated CEA levels. Approximately 10–15% of differentiated thyroid cancers metastasize to the lungs, primarily via hematogenous routes and partially through lymphatic diffusion.^157^ Nasopharyngeal carcinoma and laryngeal carcinoma may also have lung metastasis, lymphatic metastasis and hematologic metastasis. Nasopharyngeal carcinoma associated with the Epstein–Barr virus has a higher likelihood of metastasizing, and lung metastases frequently occur alongside lymph node enlargement.^158^

### 3.5 Lung cancer grafts

Lung cancer grafts can be divided into three categories based on their tissue source: patient tumor tissue, immortalized cell lines from tumor tissue, and circulating tumor cells.

#### 3.5.1 Patient tumor tissue‐derived xenotransplantation models (PDX)

**FIGURE 5:**
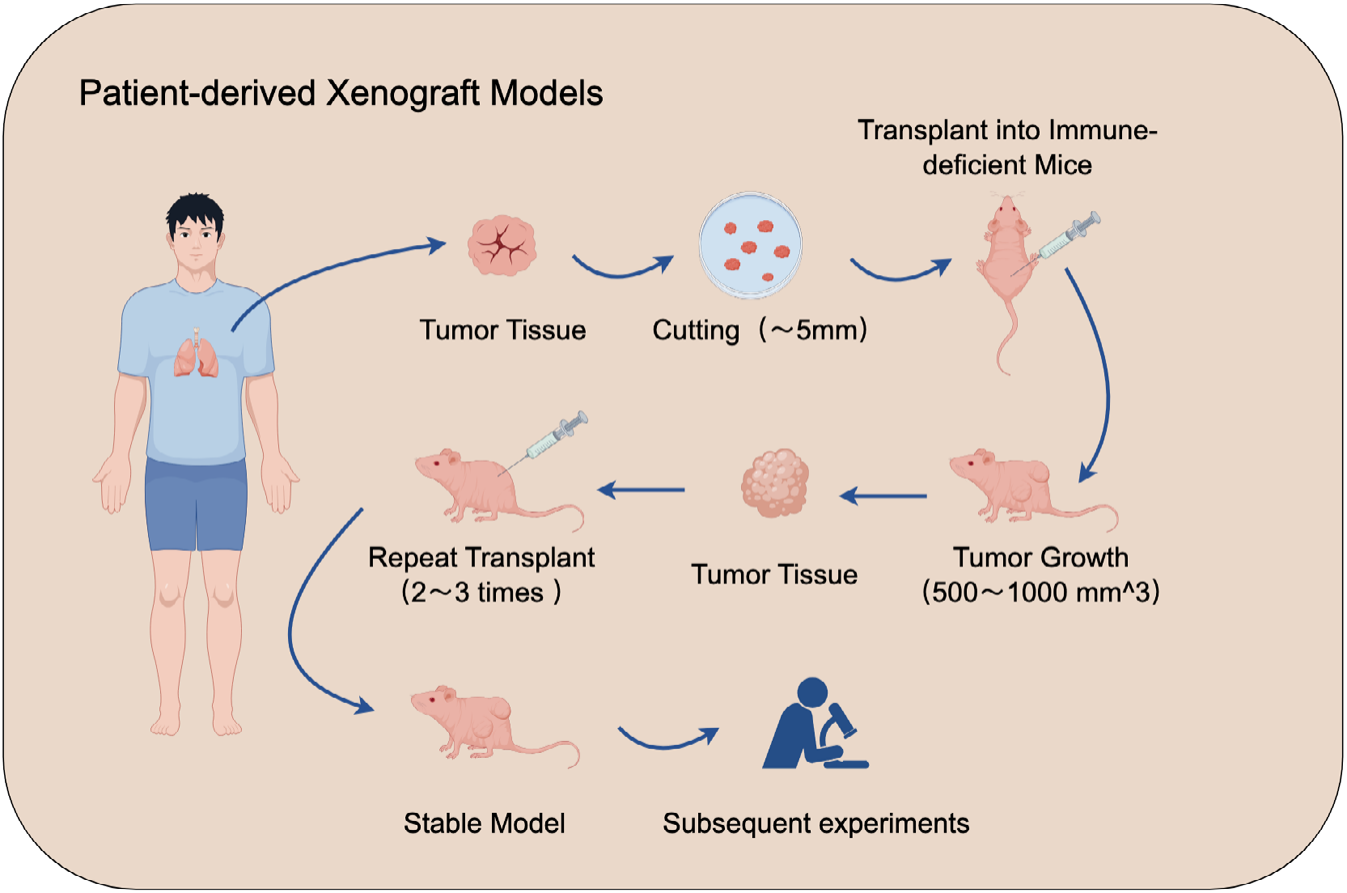
Patient‐derived xenograft models.

This model involves grafting surgically removed or biopsied tumor tissue or cell suspension into the skin or lung of highly immunodeficient mice, such as NSG, NOD/SCID, and NSFG mice, to create a tumor model.^159^ The primary graft tumor is typically removed once it has grown to 500–1000 mm^3^. This tumor is subsequently retransplanted into a new generation of mice to create a PDX model that exhibits stable growth^160^ (Figure 5). Cells derived from lung cancer tissue of patients can be cultured into three‐dimensional organoids in the lab. These organoids can subsequently be transplanted into mice to create the patient‐derived organoids‐based xenograft (PDOX).

##### Phenotypes

PDX models consistently mimic primary tumors, particularly in tissue structure, differentiation level, and marker expression. They exhibit similar growth patterns, such as acinar and solid forms, and express markers like TTF‐1 and Ki‐67.^161^, ^162^ Furthermore, PDX models preserve essential drivers of primary tumors, including EGFR, ALK, and KRAS, while showing a lower rate of genomic variation during passage.^163^, ^164^ They also accurately reproduce the microenvironmental characteristics, angiogenesis, and intercellular interactions of primary tumors, which are crucial for studying tumor biology. PDOX, when combined with circulating tumor cells (CTCs), can be used to study how CTC clusters relate to organs during tumor metastasis.

##### Applicabilities and limitations

The PDX model preserves the original tumor's genomic features, mechanisms of drug resistance, and sensitivity to drugs. This allows it to more truly reflect the biology of a patient's tumor and the response to treatment. Additionally, the PDX model is useful for studying metastasis, drug resistance, and tumor stem cells. It can also be used to screen for treatment options tailored to individual patients. However, constructing the PDX model is time‐consuming and costly, and the primary tumor's heterogeneity can affect transplantation success rates.^165^ To enhance the simulation of the primary tumor's characteristics, comprehensive sampling is essential.^166^ Mouse stroma can influence tumor behavior, and angiogenesis depends on mouse endothelial cells. This dependence complicates the realistic simulation of responses to immunotherapies, including immune checkpoint inhibitors. During tumor growth, murine‐derived fibroblasts and blood vessels gradually replace human matrix, which may affect drug permeability and microenvironment interactions.^167^, ^168^ The PDX models demonstrate their potential in drug screening.^164^

#### 3.5.2 Cell line derived xenotransplantation models (CDX)

**FIGURE 6:**
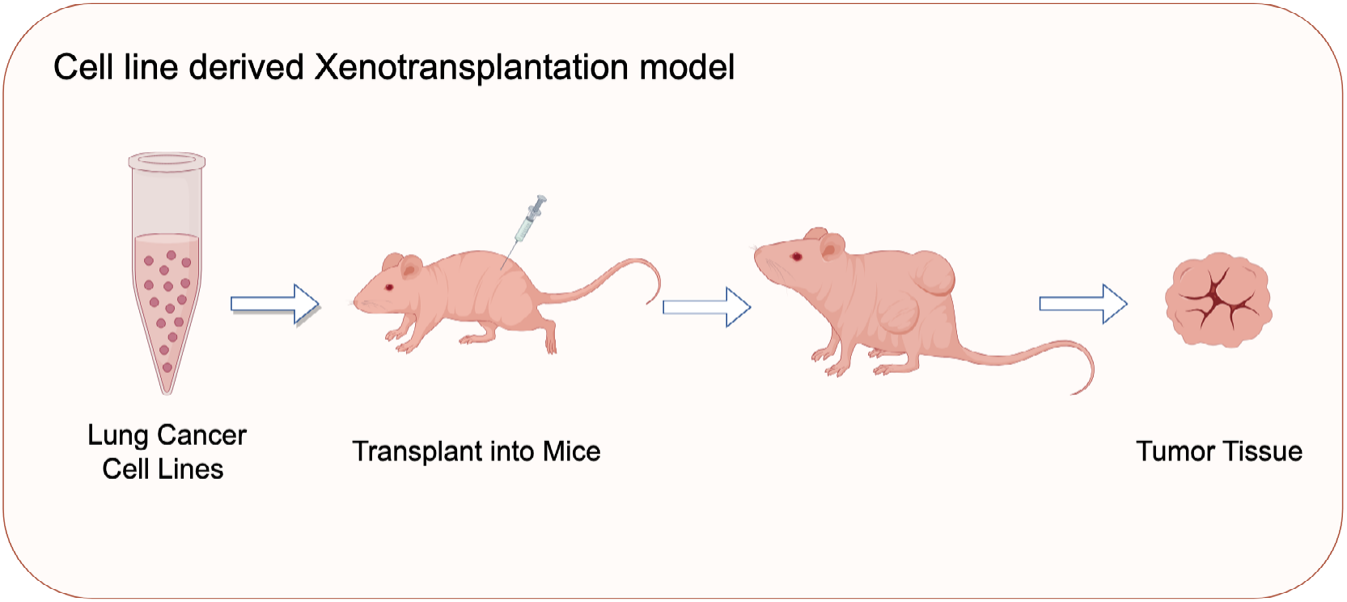
Cell line‐derived xenotransplantation models.

This model enables experimental studies by cultivating either human or mouse lung cancer cell lines (Figure 6). Cells are prompted to develop lung cancer through either orthotopic transplantation or ectopic inoculation in animals. Lung cancer cell lines can be categorized into two types: small cell lung cancer and non‐small cell lung cancer (Table 1). Human lung cancer cell lines are often implanted into nude mice, severely immunodeficient mice, or genetically engineered mice.

##### Phenotypes

Lung cancer cell lines vary in genomic characteristics and phenotypes. Small cell lung cancer cell line NCI‐H446 cells are characterized by PTEN deletion and TP53 inactivation.^169^, ^170^ BCL‐2 is highly expressed in NCI‐H209 cells and is often used to evaluate the efficacy of BCL‐2 inhibitors.^171^, ^172^ NCI‐H82 cells are suitable for constructing genetic engineering models because of MYC amplification.^173^, ^174^ DMS‐114 cells, which exhibit a high tumorigenic rate and double deletion of TP53 and RB1, are ideal for studying the transformation mechanisms of SCLC subtypes.^175^ NCI‐H1688 and A549 cells are sensitive to radiotherapy.^176^, ^177^, ^178^, ^179^ Calu‐3 cells are characterized by high expression of EGFR and amplification of HER2, and are often used to evaluate the efficacy of antibody‐coupled drugs.^180^, ^181^ EGFR mutations in HCC827, NCI‐H1650, and PC‐9 cells are useful for studying EGFR mutation‐related lung cancer.^182^, ^183^, ^184^, ^185^ Kras mutations in NCI‐H23, NCI‐H358 and NCI‐H460 cells are suitable for screening Kras targeted drugs.^184^, ^186^, ^187^, ^188^, ^189^ LLC frequently express pro‐angiogenic factors like MMP9 and VEGF, making them suitable for studying the EMT mechanisms in lung cancer.^190^, ^191^, ^192^ TC‐1 cells, after co‐transfection with HPV16 E6 and E7 genes and the ras gene, acquire tumorigenic properties.^193^ TC‐1 and SK‐MES‐1 cells are suitable for evaluating immunotherapy.^194^, ^195^

##### Applicabilities and limitations

Cell transplant‐induced lung cancer models are reproducible and straightforward to use. Researchers can manipulate the cells in controlled conditions to observe their responses to various drugs. This model has been widely used in drug screening, gene function studies, and mechanistic studies. Through gene editing and transfection of cell lines, researchers explored the roles of specific genes in lung cancer development. In addition, tumor cell lines also provide an important platform for studying tumor microenvironment, cell–cell interactions, and immune escape mechanisms. However, the genomic and transcriptomic profiles of cell lines significantly differ from those of primary tumors, potentially impacting drug response predictions.

#### 3.5.3 Xenotransplantation models derived from circulating tumor cells (CTCs)

**FIGURE 7:**
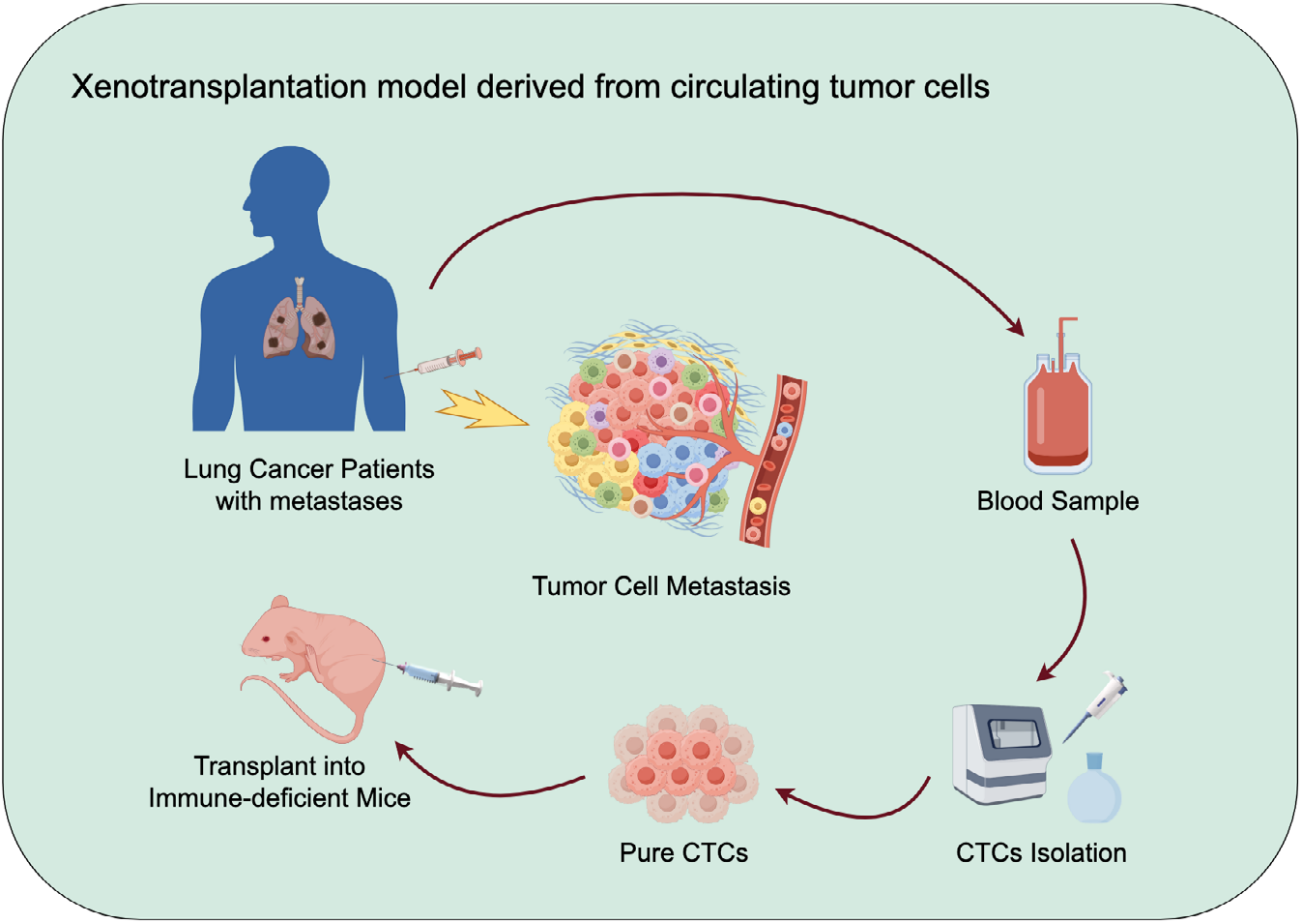
Xenotransplantation models derived from circulating tumor cells.

In this model, CTCs were isolated from the peripheral blood of lung cancer patients. Cells with high purity and activity were selected and transplanted into highly immunodeficient mice after proper identification and enrichment.^196^ Subsequently, CTCs are labeled with fluorescence or bioluminescence to track the transfer process in real time (Figure 7).

##### Phenotypes

Models created from CTCs isolated from patients with either chemotherapy‐sensitive or drug‐resistant small cell lung cancer (SCLC) can develop palpable tumors at the injection site. In some cases, micro‐metastases are also detectable, indicating that the CTCs retain their ability to proliferate. Notably, tumor growth can be observed within 4 months following transplantation.^197^ CTCs growth in animals mimics the lung cancer microenvironment, showcasing features such as angiogenesis, cell–cell interactions, and immune cell infiltration. Metastases resemble the primary tumor but may display lower levels of differentiation.^198^ The CTCs model exhibited activation of the PI3K/AKT pathway while maintaining ALK fusion, TP53 mutation, EGFR T790M, and high expression of metastasis‐related genes, including MMP9 and VEGF.

##### Applicabilities and limitations

The lung cancer model created from CTCs closely resembles the primary tumor in histology. Additionally, CTCs represent aggressive tumor subgroups with high metastatic potential. This model is more likely to develop multi‐organ metastases, making it suitable for studying metastasis mechanisms. Proliferating CTCs after transplantation may indicate the evolution of drug‐resistant clones under treatment pressure.^199^ Researchers can directly investigate hematogenous metastasis, organ colonization, and the development of drug resistance. CTCs cloning changes were tracked by liquid biopsy to guide treatment strategy adjustment, and models were constructed using patient CTCs to predict metastasis risk and drug sensitivity. However, the limited number of CTCs and the complex separation and enrichment processes require high‐precision equipment and technical support. This increases the costs of model construction and maintenance and prolongs the experimental period.

## 4 GENETICALLY ENGINEERED MOUSE MODELS (GEMMs)

**FIGURE 8:**
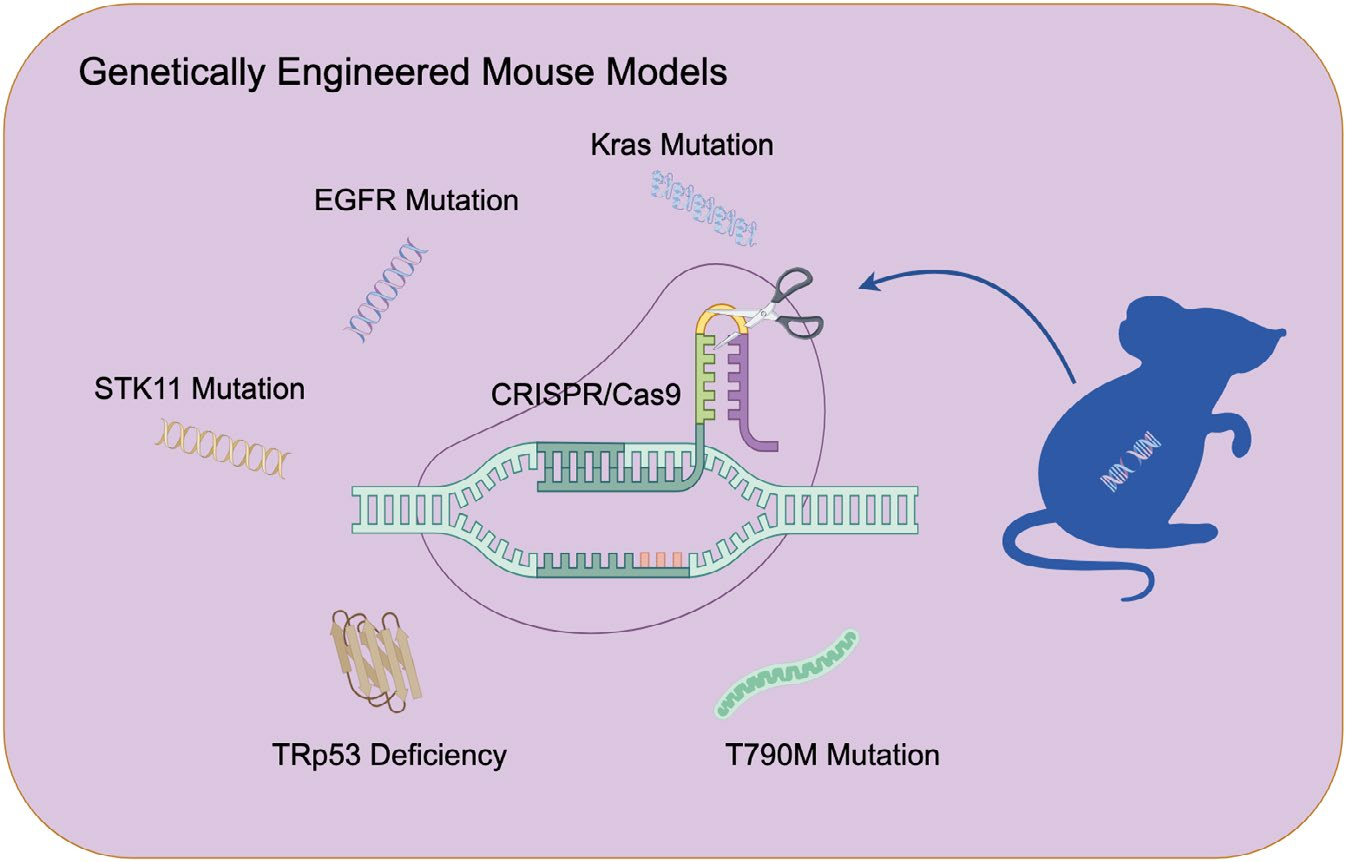
Genetically engineered mouse models.

This model utilizes CRISPR/Cas9 gene editing or transgenic technology to deliver sgRNAs and Cre recombinase into mice using adenovirus or AAV vectors (Figure 8). This process facilitates specific gene knockout or activation in the mouse genome, leading to the development of genetically engineered mouse models. Additionally, a mouse lung cancer model was created through orthotopic transplantation and tail vein injection, followed by the transplantation of tumor tissue to a new host.^200^

### 4.1 Phenotypes

The tumor tissue in the model closely resembles the original tumor morphology, exhibiting features such as cell atypia, tissue structure, and aggressiveness. The AAV‐Cre virus‐induced Kras‐LSL‐G12D model allows for significant lung tumor formation to occur within 3 months.^201^ The genetically engineered mouse model retains the mutational features of the original tumor, including KRAS and TP53, along with the corresponding phenotypic features. The Stk11/EGFP/KRAS‐LSL‐G12D model preserves the features of the KRAS mutant gene and STK11 protein deletion, making it useful for studying the multi‐gene driving mechanisms of tumors.^202^ Retaining these features makes the model valuable for investigating the genetic driving mechanisms of tumors and developing targeted therapies. This model was able to simulate the metastatic capacity of the original tumor, including transmission through the blood circulation and colonization in the lung.^203^ PC‐Cre transgenic mouse models can specifically induce mutations in Kras or EGFR in alveolar cells to trigger the development of lung cancer, and better simulate the microenvironment and tumor heterogeneity of human lung cancer.

### 4.2 Applicabilities and limitations

GEMM can simulate the development of lung cancer caused by specific gene mutations, including Kras, TP53, and EGFR, and closely matches the genetic features of human lung cancer. The tumors in the model show histopathological similarities in cell atypia, tissue structure, and aggressiveness compared to human lung cancer. Gene modification through the introduction of various genes is valuable for studying the pathogenesis, metastasis, and drug resistance mechanisms of lung cancer, as well as for evaluating the efficacy of targeted therapies like EGFR and KRAS inhibitors. GEMM also preserves the host's immune system. It can be used to study the regulatory mechanisms of the tumor immune microenvironment, including immune cell infiltration, immune escape, and angiogenesis.^204^ However, transitioning from building the model to tumorigenesis can be time‐consuming and requires specialized gene editing techniques, such as CRISPR/Cas9 and Cre‐LoxP systems, along with complex genetic manipulation. In some therapeutic contexts, GEMM may not only fail to inhibit tumors but also disrupt the function of normal cells.^205^

## 5 CONCLUSION

Animal models of lung cancer play an important role in lung cancer research. These models allow researchers to simulate the development of human lung cancer. They also help evaluate drug efficacy and explore new treatment methods. Chemically induced models create lung cancer using specific chemicals (such as tobacco smoke, ethyl carbamate, and benzopyrene). These models effectively simulate tumor development and are suitable for studying tumorigenesis and evaluating new drugs (Table 2). The orthotopic transplantation model directly implants lung cancer cells into animals. This method accurately reflects tumor growth and metastasis in their natural microenvironment, providing a solid foundation for drug screening and treatment research. The ectopic transplantation model involves transplanting lung cancer cells or tumor tissue into non‐lung tissue. This creates a controlled environment for studying tumor growth and metastasis, making it useful for evaluating new drug efficacy. The lung metastasis model has been used to investigate the impact of different injection routes, specifically the caudal vein, left ventricle, and internal carotid artery, on tumor cell metastasis, revealing significant effects on both the rate and location of metastasis. Lung metastasis models for melanoma, kidney, breast, and liver cancers are typically created using either caudal vein injection or orthotopic transplantation. These methods effectively simulate the spread of tumor cells to the lungs. Different models differ in their metastasis rates, tumor formation rates, and operational complexities. Caudal vein injection is a straightforward method that is well suited for large‐scale experiments, but it does not include a primary site. Alternatively, orthotopic transplantation provides a more accurate simulation of the natural metastasis process and is more appropriate for mechanism research and drug screening. Lung cancer modeling can be conducted using patient‐derived xenografts (PDX), genetically engineered mouse models (GEMM), and cell line transplantation models. The PDX model maintains the genomic features of the primary tumor, making it suitable for personalized treatment. GEMM simulates lung cancer caused by specific gene mutations. This model is ideal for investigating genetic mechanisms and targeted therapies. The cell line transplantation model is highly reproducible and commonly used for drug screening and mechanism research. However, its genomic characteristics differ significantly from those of primary tumors. The circulating tumor cell model is particularly useful for studying the mechanisms of metastasis, although it requires invasive procedures to obtain samples.

**TABLE 2:** Cell strains of lung cancer.

Source	Types of lung cancer	Cell Strain	Methods	Phenotypes	Application	References
Human	SCLC	NCI‐H446	5 × 10^6^ cells in 0.1 mL of PBS mixed with Matrige (s.c.)	TP53 inactivation, PTEN deletion, typical neuroendocrine markers (Synaptophysin+, CD56+)	Sensitivity tests for topoisomerase inhibitors such as irinotecan	[169, 170]
		NCI‐H209	1 × 10^7^ cells/mL in 1:1 ratio of PBS mixed with Matrige (s.c.)	It carries high expression of BCL‐2 and is sensitive to apoptosis inducers	Apoptosis pathway and drug screening targeting BCL‐2	[171, 172]
		NCI‐H82	1 × 10^5^ cells in 300 μL containing serum‐free RPMI‐1640 and 300 μL Matrigel (s.c.)	Suspension growth, MYC amplification, Etoposide resistance	Because of MYC amplification, NCI‐H82 is suitable for CRISPR screening research, drug resistance mechanism and MYC targeted therapy research	[173, 174]
		DMS‐114	3 × 10^6^ cells in 250 μL of PBS (s.c.)	Adherent growth, TP53/RB1 double deletion, Low expression of neuroendocrine markers	Study on the transformation mechanism of SCLC subtype with high tumor formation rate	[175]
		NCI‐H1688	1 × 10^6^ cells/mL (i.t.)	Homozygous loss of Rb1 gene, neuroendocrine differentiation, sensitivity to radiotherapy (G2/M phase block after irradiation is significant)	DNA damage repair mechanism and radiotherapy sensitization strategy	[176, 177]
	NSCLC	A549	1 × 10^6^ to 1 × 10^7^ cells/mL (s.c.)	Adherent growth, moderate sensitivity to cisplatin, KRAS G12S mutation, TP53 wild type	Chemotherapy sensitivity, EMT mechanism, KRAS signaling pathway study, subcutaneous tumor formation rate is high	[178, 179]
		Calu‐3	5 × 10^5^ to 5 × 10^6^ cells/mouse (s.c.)	EGFR was highly expressed and HER2 was amplified	Evaluation of the efficacy of antibody‐coupling drugs such as T‐DM1	[180, 181]
		HCC827	1 × 10^7^ cells/mouse (s.c.)	EGFR exon19 deletion, Sensitive to EGFR‐TKI, Used to study EGFR mutation‐associated lung cancer	It is often used to study EGFR mutation‐associated lung cancer, EGFR‐TKI (gefitinib/ocitinib) sensitivity and mechanism of resistance	[182]
		NCI‐H1299	1 × 10^6^ cells/mouse (s.c.)	Deletion of p53 gene	Research on apoptosis mechanisms (e.g. screening of BCL‐2 inhibitors)	[206]
		NCI‐H1650	5 × 10^6^ cells in 100 μL of PBS (s.c.)	EGFR exon19 deletion, PTEN deletion, High expression of interstitial markers, Primary resistance to EGFR‐TKI (gefitinib)	Correlation analysis of EMT (epithelial‐mesenchymal transformation) and targeted therapy, mechanism study of GFR‐TKI resistance (PTEN deletion /MET amplification mediated bypass activation)	[183, 184]
		NCI‐H1975	2 × 10^6^ cells in 0.2 mL of PBS mixed with 0.2 mL Matrige (s.c.)	EGFR L858R + T790M double mutation, Constitutive activation of PI3K/AKT pathway (high AKT phosphorylation level), Sensitive to ocitinib (third generation EGFR‐TKI)	Study on the efficacy and resistance mechanism of the third generation EGFR‐TKI (such as ocitinib)	[185]
		NCI‐H23	5 × 10^6^ cells in 100 μL PBS (s.c.)	KRAS mutation	Evaluation of the efficacy of Pachymic acid	[186, 187]
		NCI‐H358	8 × 10^6^ to 2 × 10^7^ cells in 200 μL of PBS (s.c.)	KRAS mutation	Studies of KRAS G12C‐specific inhibitors such as Sotorasib	[184, 188]
		NCI‐H460	4 × 10^6^ cells/mL (s.c.)	KRAS mutation, Sensitive to mTOR inhibitors	Studies of metabolic reprogramming (e.g., glutamine dependence) have shown a strong propensity to transfer	[189]
		NCI‐H1975	1 × 10^6^ cells in 0.1 mL PBS (s.c.)	EGFR L858R + T790M double mutation	Validation of the efficacy of third generation EGFR‐TKI (oxitinib)	[162, 207]
		PC‐9	1 × 10^6^ cells in 200 μL of PBS (s.c.)	Expression of EGFR mutation (exon 19 deletion)	Construction of acquired drug resistance model of T790M	[208]
		SK‐MES‐1	2 × 10^6^ cells/mL (s.c.)	Expression of PD‐L1	Regulation of immune checkpoint (PD‐L1) expression	[194]
Mouse	NSCLC	Lewis Lung Carcinoma (LLC)	5 × 10^6^ to 2 × 10^7^ cells/mL (s.c.)	Expression of VEGF, MMP‐9 and other pro‐angiogenic factors, Sensitive to methotrexate	Chemotherapy drugs (such as cisplatin), anti‐angiogenesis, metastasis mechanism treatment	[190, 191, 192]
		CMT167	1 × 10^6^ cells in 100 μL of PBS (s.c.)	Sensitive to MEK inhibitors	Study on the mechanism of KRAS signaling pathway targeting therapy	[209]
	Genetic engineering model	TC‐1	5 × 10^5^ cells/mouse (IV)	Expression of HPV16 E7 antigen, Induce immune clearance response	Used in vaccine research, immunotherapy (such as PD‐1 antibody) efficacy evaluation	[195]

Abbreviations: i.t., intratracheal instillation; IV, tail vein injection; s.c., subcutaneous injection.

The various animal models described are valuable for studying the biological characteristics and mechanisms of lung cancer, However, these models also have limitations. No single model can represent all the characteristics of human lung cancer, Thus, selecting the right model, which can simulate the clinical manifestations of lung cancer, is vital for understanding lung cancer development and treatment options, and laying the groundwork for future diagnosis and treatment of the disease.^210^

## AUTHOR CONTRIBUTIONS

**Zixuan Yang:** Conceptualization; data curation; investigation; methodology; project administration; writing – original draft; writing – review and editing. **Xianbin Zhao:** Conceptualization; formal analysis; investigation; methodology; visualization; writing – original draft. **Lili Tan:** Investigation; methodology; validation. **Pingxinyi Que:** Data curation; investigation; methodology. **Tong Zhao:** Investigation; methodology; visualization. **Wei Huang:** Conceptualization; formal analysis; supervision; validation. **Dejiao Yao:** Conceptualization; funding acquisition; writing – review and editing. **Songqi Tang:** Conceptualization; supervision; visualization; writing – review and editing.

## FUNDING INFORMATION

This work was supported by the National Natural Science Foundation of China (grant number 82474436) and the Sichuan Provincial Administration of Traditional Chinese Medicine (grant number 2023MS564).

## ETHIC STATEMENT

This review article is grounded in ethical principles, ensuring the accurate and objective presentation of information. All sources are properly credited, and potential biases are carefully minimized to maintain a fair and transparent exploration of the topic.

## CONFLICT OF INTEREST STATEMENT

The authors declare that they have no known competing financial interests or personal relationships that could have appeared to influence the work reported in this paper.
